# Machine learning-assisted analysis of serum metabolomics for identifying biomarkers in intrinsic and idiosyncratic drug-induced liver injury

**DOI:** 10.3389/fphar.2025.1727462

**Published:** 2026-02-27

**Authors:** Xianni Wei, Jinbao Wei, Yuhong Huang, Siheng Lian

**Affiliations:** Department of Pharmacy, Haicang Hospital Affiliated to Xiamen Medical College, Xiamen, Fujian, China

**Keywords:** metabolomics, drug-induced liver injury, intrinsic, idiosyncratic, biomarkers, machine learning

## Abstract

**Objective:**

This project aims to employ high-performance chemical isotope labeling (HP-CIL) liquid chromatography–mass spectrometry (LC-MS) to conduct a metabolomic study on the mechanisms underlying intrinsic and idiosyncratic drug-induced liver injury (DILI). By comparing the metabolic characteristics between these two types of DILI, we seek to identify biomarkers for predicting intrinsic and idiosyncratic DILI using machine learning strategies.

**Methods:**

Based on the diagnostic criteria outlined in the EASL clinical practice guidelines on drug-induced liver injury, a review published in NEJM, enrolled DILI cases were classified according to the pathogenic mechanism into an intrinsic type (n = 17) and an idiosyncratic type (n = 27). Serum samples were collected from both groups. Metabolomic profiling was performed using high-performance chemical isotope labeling liquid chromatography–mass spectrometry (HP-CIL LC-MS) to identify differentially expressed metabolites between the two groups. Metabolites that showed significance in both univariate and multivariate statistical analyses were selected for further receiver operating characteristic (ROC) analysis. Machine learning approaches were employed to develop diagnostic models for distinguishing intrinsic and idiosyncratic DILI. These models were compared to identify potential biomarkers capable of discriminating between the two types of DILI, and the diagnostic performance of these candidate biomarkers was evaluated.

**Result:**

Serum metabolomic profiling identified four differential metabolites that distinguished intrinsic from idiosyncratic DILI through multivariate and univariate statistical analyses, followed by ROC curve analysis and machine learning-based selection. These potential biomarkers included Alanyl-Glycine (level 1),N2-Acetyl-L-Cystathionine (level 2a), Isomer 1 of 5-Hydroxyindoleacetic acid (level 2a), and Isomer 1 of 5-Hydroxyindoleacetic acid (level 2a). ROC analysis using multiple machine learning models yielded area under the curve (AUC) values greater than 0.8 for all models, indicating high diagnostic performance. Under a multivariate regression model, internal cross-validation (CV) within the training set demonstrated robust model tuning and stability, with an AUC of 0.983. Holdout validation further confirmed model reliability with an AUC of 0.935. Metabolic pathway analysis of these metabolites revealed that the most significantly associated pathways affecting intrinsic and idiosyncratic DILI were primarily related to amino acid metabolism, including tryptophan metabolism, tyrosine metabolism, cysteine and methionine metabolism, and the biosynthesis of phenylalanine, tyrosine, and tryptophan.

**Conclusion:**

This study demonstrates that machine learning-assisted serum metabolomics can effectively characterize currently well-established intrinsic and idiosyncratic drug-induced liver injury, reveal metabolic disparities between the two types, and identify differential metabolites associated with their respective pathogenesis. These findings provide a valuable reference for predicting the mechanistic type of liver injury induced by various hepatotoxic drugs in the future.

## 1 Introduction

Drug-induced liver injury (DILI) has attracted increasing attention as it is not only one of the common causes of clinical liver diseases—particularly acute liver injury—but also one of the most frequent reasons for drug development failure and post-marketing withdrawal (Edwards et al., 2023). The pathogenesis of DILI is complex, involving multiple factors related to the drug, the host, and even environmental influences (Skat-Rordam et al., 2025). It often results from the sequential or synergistic effects of multiple mechanisms, which have not yet been fully elucidated (Drug-Induced Liver Injury Group et al., 2015).

Clinical guidelines broadly classify the mechanisms of DILI into two categories: intrinsic and idiosyncratic. Intrinsic hepatotoxicity refers to the innate damage caused by a drug and/or its metabolites to the liver, which is usually dose-dependent, predictable, has a short latency period, and shows little interindividual variability. Intrinsic hepatotoxicity may further trigger secondary mechanisms of liver injury, such as immune and inflammatory responses (Drug-Induced Liver Injury Group et al., 2015).

In contrast, idiosyncratic hepatotoxicity is unpredictable, exhibits significant individual variation, and is not clearly correlated with drug dosage, route of administration, or treatment duration. It is difficult to replicate in animal models and manifests with diverse clinical presentations (Drug-Induced Liver Injury Group et al., 2015).

Conventional animal toxicology studies can typically detect intrinsic hepatotoxicity, whereas current preclinical models fail to predict idiosyncratic DILI, as its occurrence involves both genetic and non-genetic factors related to human immune and metabolic pathways. Therefore, idiosyncratic hepatotoxicity is generally identifiable only during clinical trials. However, due to the limited sample size in clinical trials, idiosyncratic DILI—which often has a very low incidence—is frequently identified only after a drug has been marketed (National Medical Products Administration Center for Drug Evaluation, 2023).

A review published in NEJM in 2019 proposed a novel pathogenic mechanism termed “indirect” DILI, which is thought to arise from the pharmacological effects of a drug itself rather than from its intrinsic hepatotoxicity or immunogenicity (Hoofnagle and Björnsson, 2019). This form of DILI typically occurs in susceptible populations, such as individuals with underlying liver conditions, where the drug alters the physiological state, thereby inducing liver injury or exacerbating pre-existing liver disease. However, the concept of indirect DILI currently lacks support from relevant clinical and experimental evidence (Bonkovsky et al., 2018). Some researchers argue that its definition and scope remain inadequately defined (Li et al., 2024). Indirect DILI is generally not dose-dependent and shares certain similarities with idiosyncratic DILI, making practical distinction between the two challenging (Fontana et al., 2023).

Currently, it remains impossible to distinguish DILI mechanisms based solely on clinical biochemical parameters or pathological features. The classification of DILI into specific mechanistic types—caused by various hepatotoxic drugs—relies primarily on empirical consensus among experts. Whether intrinsic and idiosyncratic DILI exhibit clear distinctions still lacks thorough and systematic characterization through objective experimental indicators. Understanding the type of DILI mechanism involved could provide valuable reference points for its treatment.

Recently, metabolomic approaches have led to the discovery of novel serum biomarkers—such as glutamate dehydrogenase, high mobility group box 1 (HMGB1), and microRNA-122—which offer important insights for the diagnosis and prognostic assessment of DILI (Thulin et al., 2017; Howell et al., 2018; Church et al., 2019).

High-performance chemical isotope labeling (HP-CIL) has emerged as a highly effective strategy to enhance both the coverage and quantitative accuracy of metabolomic analyses. This method significantly improves metabolite detection rates and enables more precise quantification of low-abundance metabolites (Han and Li, 2018).

This study applies HP-CIL LC-MS-based metabolomics to characterize well-established cases of intrinsic and idiosyncratic drug-induced liver injury (DILI), aiming to uncover differences in metabolic profiles between these two types. By integrating machine learning strategies, we further identify differential metabolites associated with each pathogenic mechanism. These findings provide a valuable reference for elucidating the mechanistic basis of liver injury induced by various hepatotoxic drugs.

## 2 Materials and methods

### 2.1 Study population and sample collection

Serum samples were collected from patients diagnosed with drug-induced liver injury (DILI) and hospitalized between October 2022 and October 2024. Based on the Chinese Guidelines for the Diagnosis and Treatment of Drug-Induced Liver Injury (Drug-Induced Liver Injury Group et al., 2015), the following inclusion criteria were applied.

1. History of suspected drug exposure prior to the onset of liver injury;
2. Liver injury indicators meeting the diagnostic criteria for DILI;
3. Exclusion of other liver diseases—including viral hepatitis (types A, B, C, and E, as well as other hepatotropic viruses such as CMV, HSV, and EBV), alcoholic liver disease, and autoimmune liver disease—through comprehensive medical history and laboratory tests;
4. The mechanism of liver injury induced by the drug is consistent with currently well-established classifications of intrinsic or idiosyncratic DILI (Hoofnagle and Björnsson, 2019; European association for the study of the liver, 2019; Yu and Chen, 2021).

Exclusion criteria were as follows.

1. Unclear history of drug exposure;
2. Inability to rule out liver injury due to other causes;
3. Patients with liver failure;
4. Cases where the drug is known to cause both intrinsic and idiosyncratic DILI according to current consensus.

A total of 44 patients who met the aforementioned criteria were enrolled, including 17 cases of intrinsic DILI and 27 cases of idiosyncratic DILI. Blood samples were left at room temperature for 0.5 h before centrifugation at 4 °C for 10.0 min at 3,000 rpm to separate the upper serum layer. Serums were transferred and stored at −80 °C until use.

This study was approved by the Medical Ethics Committee of Xiamen Haicang Hospital (Approval No. KY-2022008).

### 2.2 Patient information collection

Gender, age, and liver function parameters at the time of admission were collected from the hospital information system for both DILI patients and control subjects. Liver function indicators included alanine aminotransferase (ALT), aspartate aminotransferase (AST), alkaline phosphatase (ALP), gamma-glutamyl transferase (GGT), and total bilirubin (TBIL). For DILI patients, the names of the suspected causative drugs were also documented.

### 2.3 Untargeted metabolomics analysis

Untargeted metabolomics was employed to analyze serum samples from patients with intrinsic and idiosyncratic types of DILI, aiming to identify differential metabolites between the two groups.

#### 2.3.1 Sample preparation

Frozen serum samples were thawed and thoroughly mixed using a vortex mixer for 30 s. Subsequently, the samples were centrifuged at 4 °C for 10 min at 12,000 r/min (with a centrifugal radius of 10 cm). Before mass spectrometry analysis, the proteins in the serum needed to be removed. Three times the volume of methanol (Fisher Chemical, Waltham, MA, United States) was added to the serum, vortexed and shaken for 30 s, and then placed at −20 °C for 1 h. The supernatant was centrifuged at 15,000 rpm for 30.0 min at 4 °C and dried in a refrigerated CentriVap concentrator system (Labconco, Kansas City, MO, United States) to obtain the powder containing the serum metabolite, which was stored at −80 °C. In addition, 38.0 μL of serum was aspirated in each sample, mixed, and used as a pooled sample. The metabolite powder of the pooled sample was obtained by the same operation and stored at − 80 °C.

#### 2.3.2 Sample labeling

Each aliquoted sample underwent secondary metabolome labeling for amine/phenol subclasses. After drying under a nitrogen concentrator, the samples were reconstituted in 25 µL of mass spectrometry-grade water. The labeling procedure strictly followed validated isotopic labeling metabolomic protocols using a standardized, methodologically established workflow. Specifically, 12.5 µL of buffer reagent A and 37.5 µL of ^12^C-labeled reagent B.12 (for individual and pooled samples) or ^13^C-labeled reagent B.13 (for pooled samples only) were added to each sample. The mixture was vortexed and incubated at 40 °C for 45 min. After incubation, 7.5 µL of reagent C was added to quench the excess labeling reagent, followed by incubation at 40 °C for 10 min. Finally, 30 µL of pH-adjusting reagent D was introduced.

#### 2.3.3 Liquid chromatography-mass spectrometry analysis

The labeled samples were analyzed using an Agilent 1,290 ultra-performance liquid chromatography (Thermo Scientific, Waltham, MA, United States) coupled with a 6,546 quadrupole-time-of-flight mass spectrometer (Bruker, Billerica, MA, United States). The mobile phase A (MPA) was 0.1% formic acid in water (v/v), and mobile phase B (MPB) was 0.1% formic acid in acetonitrile (v/v). The chromatographic gradient elution conditions were as follows: t = 0 min, 25% B; t = 10.0 min, 99% B; t = 13.0 min, 99% B; t = 13.1 min, 25% B; t = 16.0 min. The flow rate was 400.0 μL/min. All MS spectra were collected in positive ion mode at a spectral acquisition rate of 1 Hz. Before analyzing the samples, the quality control (QC) samples were repeatedly tested at least 5 times until the signal stabilized. During LC-MS analysis, the individual samples were arranged in a random sequence, interspersed with a blank sample, a12C-labeled standard sample, and a QC sample every 15 random samples.

This study employed positive ionization mode for mass spectrometry detection based on the following key rationales: 1) The High-Performance Chemical Isotope Labeling (HP-CIL) technique utilized in this research targets four major metabolite classes–amines/phenols, carboxyls, hydroxyls, and carbonyls–using dedicated tagging reagents such as dansyl chloride and DmPA bromide. These reagents incorporate strong ionization-enhancing groups (e.g., dimethylamino, pyridinium), which convert neutral or weakly ionizable metabolites into derivatives that ionize efficiently in positive mode, enhancing detection sensitivity by 10- to 1000-fold (Zhao et al., 2019). 2) The labeling reactions mitigate inherent polarity differences among metabolites, enabling compounds typically targeted in negative mode–such as organic acids and fatty acids–to yield more stable and robust responses in positive ionization mode (Guo et al., Anal. Chim. Acta, 2016). 3) Using a single positive ionization mode eliminates the need for polarity switching, thereby reducing overall analysis time and preventing signal dispersion. Combined with the four-channel labeling strategy, this approach covers 86%–96% of endogenous human metabolites (Zhao et al., 2019), ensuring comprehensive metabolome coverage. Experimental validation confirmed that the peak intensity relative standard deviation (RSD) for target metabolites (e.g., amino acids, organic acids, glutathione derivatives) was below 10%, meeting the requirements for reliable quantitative analysis.

### 2.4 Data analysis

Statistical analysis of patient characteristics was performed using SPSS version 26.0 (IBM, United States). Normally distributed continuous variables are presented as mean ± standard deviation and compared using the Student’s t-test. Non-normally distributed continuous variables are expressed as median (Q1, Q3) and compared using the Mann–Whitney U test. Categorical variables are described as frequency and percentage (%), and group comparisons were conducted using the chi-square test or Fisher’s exact test, as appropriate. A P-value <0.05 was considered statistically significant.

After data acquisition and export, all data were imported into IsoMS Pro version 1.3.2 for processing and analysis. Metabolites in serum samples from the intrinsic and idiosyncratic DILI groups were compared using two analytical approaches. A three-tiered metabolite identification strategy was applied to structurally annotate detected metabolites.

Statistical power was assessed *post hoc* using the observed effect sizes of the identified metabolites. Cohen’s d values were calculated via the Psychometrica online tool (https://www.psychometrica.de/effect_size.html) to determine the effect size for each key differential metabolite. Subsequently, a power analysis was performed with G*Power software (version 3.1) based on the smallest observed Cohen’s d, the pre-set alpha level of 0.05, and the actual sample sizes.

#### 2.4.1 Multivariate statistical analysis

Partial least squares-discriminant analysis (PLS-DA), a supervised method, was employed to discriminate metabolic profiles between groups. The model’s robustness was evaluated through permutation testing. *R* ^2^, representing the goodness of fit, was considered acceptable when >0.5. Q^2^, indicating predictive ability, was deemed adequate when >0.5 and excellent when >0.9. Variable importance in projection (VIP) values were derived from the PLS-DA model; metabolites with VIP >1 were regarded as significant. A heatmap was generated to display the top 30 VIP metabolites. Metabolites identified at levels 1 and 2 were considered outcomes of the multivariate analysis.

#### 2.4.2 Univariate statistical analysis

Following univariate differential analysis of all detected metabolites, those satisfying the criteria of FC > 1.2 or <0.83 with a raw P-value <0.05 were defined as significantly differential metabolites. It should be noted that these P-values were not adjusted for multiple testing, as this study is exploratory in nature and aims to maximize the discovery of potential biomarkers while avoiding overly conservative criteria that might lead to missed signals.

To succinctly present the core results in the main text, key metabolites were identified through a refined screening process: first, all significant metabolites were ranked by ascending adjusted p-value to prioritize statistical reliability; second, only those with confident Level 1 or 2 identifications were retained to ensure biological interpretability, thereby excluding unidentified Level 3 features; finally, the top 15 upregulated and top 15 downregulated metabolites from this filtered list were selected for inclusion in Table 1. The overall changes (up/downregulation) and significance of these metabolites between the intrinsic and idiosyncratic DILI groups were visualized using a volcano plot. The complete list of all significant metabolites, including Level 3 features, is available in Supplementary Table S2.

**TABLE 1:** Top 30 primary and secondary metabolites with significant difference Intrinsic versus Idiosyncratic DILI.

No.	Molecular weight	Metabolite	Fold change	P-value	Identification level
Top 10 upregulated					
A-T543	146.0691	Alanyl-Glycine	1.446	4.86E-05	Level 1
A-950	142.0378	5-Hydroxymethyluracil	1.279	2.11E-04	Level 1
A-T2493	155.0695	Histidine	1.222	3.03E-04	Level 1
A-1714	191.0614	Isomer 1 of 5-Hydroxyindoleacetic acid (level 2a)	1.231	3.75E-04	Level 2a
A-T720	224.0797	3-Hydroxy-L-kynurenine	1.325	3.85E-04	Level 2a
A-T2630	252.1222	Prolyl-Histidine	1.376	4.91E-04	Level 1
A-T691	202.0954	Prolyl-Serine	1.295	5.34E-04	Level 1
A-984	117.0426	L-2-amino-3-oxobutanoic acid	1.381	6.13E-04	Level 2a
A-T1005	161.0688	N-Methyl-L-glutamic acid	1.297	9.00E-04	Level 2a
A-T1765	217.1426	gamma-L-Glutamylputrescine	1.350	9.16E-04	Level 2a
A-709	117.0788	Isomer 1 of 5-Aminopentanoic acid	1.259	9.38E-04	Level 2a
A-1993	196.0734	Isomer 1 of 5-Hydroxyindoleacetic acid (level 2a)	10.103	1.25E-03	Level 2a
A-T2312	267.0968	Deoxyguanosine	1.409	1.29E-03	Level 1
A-1466	89.0478	Isomer 2 of N-Methyl-Glycine	1.317	2.03E-03	Level 2a
A-T714	264.078	N2-Acetyl-L-Cystathionine	1.524	2.21E-03	Level 2a
Top 10 downregulated					
A-1889	152.0474	3-Methylsalicylic acid	0.394	3.31E-05	Level 1
A-T331	245.1012	Hydroxyprolyl-Asparagine	0.521	5.27E-05	Level 2a
A-T2235	152.0473	4-Hydroxyphenylacetic acid	0.341	5.31E-05	Level 1
A-T242	245.1012	Asparaginyl-Hydroxyproline	0.291	9.67E-05	Level 2a
A-T1202	174.1004	Glycyl-Valine	0.297	1.02E-04	Level 1
A-T145	276.1321	Saccharopine	0.403	1.39E-04	Level 1
A-1146	175.048	Isomer 2 of 7-Cyano-7-carbaguanine	0.380	1.89E-04	Level 2a
A-1489	262.0947	Isomer 1 of N-Acetyl-5-Hydroxy-L-tryptophan	0.460	2.54E-04	Level 2a
A-T2760	174.1004	Gly-Norvaline	0.356	2.66E-04	Level 1
A-955	176.0314	Isomer 1 of Ascorbic acid	0.437	3.23E-04	Level 2a
A-T2190	152.0473	2-Hydroxyphenylacetic acid	0.301	3.49E-04	Level 1
A-T342	204.0746	Glutamyl-Glycine	0.513	4.95E-04	Level 1
A-T1781	128.0473	Dihydrophloroglucinol	0.475	8.14E-04	Level 2a
A-851	175.0479	Isomer 1 of 7-Cyano-7-carbaguanine	0.539	8.68E-04	Level 2a
A-T464	133.0375	Aspartic acid	0.634	8.85E-04	Level 1

The p-values presented are raw (unadjusted) p-values.

#### 2.4.3 Receiver operating characteristic (ROC) curve analysis

MetaboAnalyst (https://www.metaboanalyst.ca) was used to compare metabolite profiles between the intrinsic and idiosyncratic DILI groups. ROC analysis was applied to evaluate the discriminative power of common metabolites that were highlighted in both multivariate and univariate analyses. The area under the curve (AUC) was used to assess the diagnostic potential of each metabolite. An AUC >0.7 was considered indicative of diagnostic value. Several machine learning methods—including support vector machine (SVM), multiple regression, linear regression, and random forest—were implemented via MetaboAnalyst to develop diagnostic models discriminating intrinsic from idiosyncratic DILI. Performance among these techniques was compared. Metabolites selected through these approaches were considered potential biomarkers for DILI. The training set comprised 60% of the data from each group, with the remaining data used as the test set. Model accuracy was evaluated based on predictions within the test set.

The differentially expressed metabolites identified from univariate analysis (fold change >1.2 or <0.83, adjusted p < 0.05) were further filtered to retain only those confirmed via a three-level identification strategy—specifically, Level 1 (exact match to chemical standards or reference spectra from authoritative databases such as HMDB or METLIN) and Level 2 (match to MS/MS spectra or fragment ion information in databases). This process yielded a total of 174 qualified metabolites (75 Level 1 and 99 Level 2). Pathway enrichment analysis was subsequently performed on these Level 1 and Level 2 metabolites using the Pathway Analysis module of the MetaboAnalyst platform (www.metaboanalyst.ca). The Global Test algorithm was applied for enrichment analysis, while topological analysis utilized relative-betweenness centrality. The KEGG *Homo sapiens* metabolic pathway database served as the reference pathway library.

#### 2.4.4 Sensitivity analysis

To address the potential confounding effect of underlying malignancy, a sensitivity analysis was performed by excluding the eight patients with liver injury induced by anticancer chemotherapeutic agents. The key multivariate model (OPLS-DA) and biomarker performance were re-evaluated on the subset of patients (n = 36) with DILI not related to cancer.

The identification of metabolites adhered to the guidelines established by the Metabolomics Standards Initiative (MSI) (Sumner et al., Metabolomics 2007). In accordance with the MSI guidelines, the confidence levels for metabolite identification are categorized into four tiers:

Level 1 (Identified Compounds): Confirmed by matching a minimum of two independent and orthogonal data types against an authentic chemical standard analyzed under identical experimental conditions within the same laboratory. In this study, this included matching accurate mass (mass error <5 ppm), chromatographic retention time (RT deviation <0.2 min), and isotopic distribution pattern.

Level 2 (Putatively Annotated Compounds): Based on spectral similarity to public or commercial spectral libraries without reference to an authentic standard under the same experimental conditions (e.g., matching by accurate mass and MS/MS spectrum from libraries).

Level 3 (Putatively Characterized Compound Classes): Classified based on characteristic physicochemical properties or spectral similarity to a known class of compounds (e.g., lipids, flavonoids).

Level 4 (Unknown Compounds): Distinguished solely by analytical features such as mass-to-charge ratio and retention time, remaining uncharacterized and unidentified.

### 2.5 Clinical characteristics of the patients

Among the 44 enrolled DILI patients, cases were categorized based on the mechanism of liver injury. The intrinsic group (n = 17) included acetaminophen (n = 5), anticancer chemotherapeutic agents (n = 8), valproate sodium (n = 3), and cyclosporine (n = 1). The idiosyncratic group (n = 27) consisted of macrolides (n = 1), fluoroquinolones (n = 4), cephalosporins (n = 2), sulfonamides (n = 3), tetracyclines (n = 2), phenytoin sodium (n = 13), and fenofibrate (n = 2).

The intrinsic group comprised 17 patients, including 4 males and 13 females, with a mean age of 57.18 ± 17.02 years. The median values (Q1, Q3) of ALT, AST, ALP, GGT, and TBil were 115.20 (80.00–213.83) U/L, 97.20 (60.13–169.68) U/L, 126.00 (98.25–178.30) U/L, 128.80 (73.40–303.35) U/L, and 13.13 (8.94–20.63) µmol/L, respectively. The idiosyncratic group included 27 patients, with 21 males and 6 females, and a mean age of 60.11 ± 16.53 years. No statistically significant difference was observed in age between the two groups (t = −0.567, P = 0.574). However, a significant difference was found in gender distribution (χ^2^ = 12.51, P < 0.001).

### 2.6 Serum metabolomics analysis

#### 2.6.1 Multivariate statistical analysis

**FIGURE 1:**
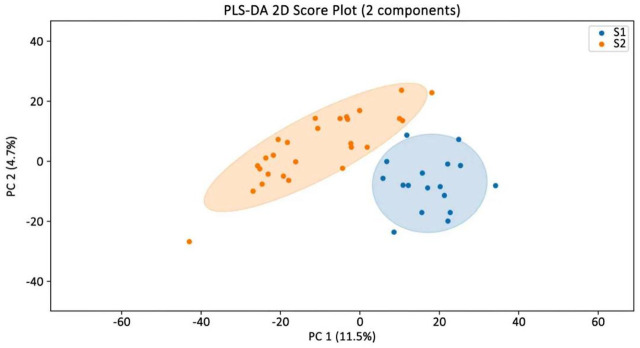
PLS-DA analysis of the intrinsic DILI group versus the idiosyncratic DILI group.

Partial Least Squares-Discriminant Analysis (PLS-DA), a supervised multivariate statistical method, was applied to analyze the metabolomic data obtained from the serum samples. The results are presented in Figure 1. The PLS-DA score plot showed clear separation between the intrinsic DILI group (Group S1, blue) and the idiosyncratic DILI group (Group S2, orange), with distinct clustering in different regions. This indicates significant differences in metabolite profiles between the two types of DILI.

**FIGURE 2:**
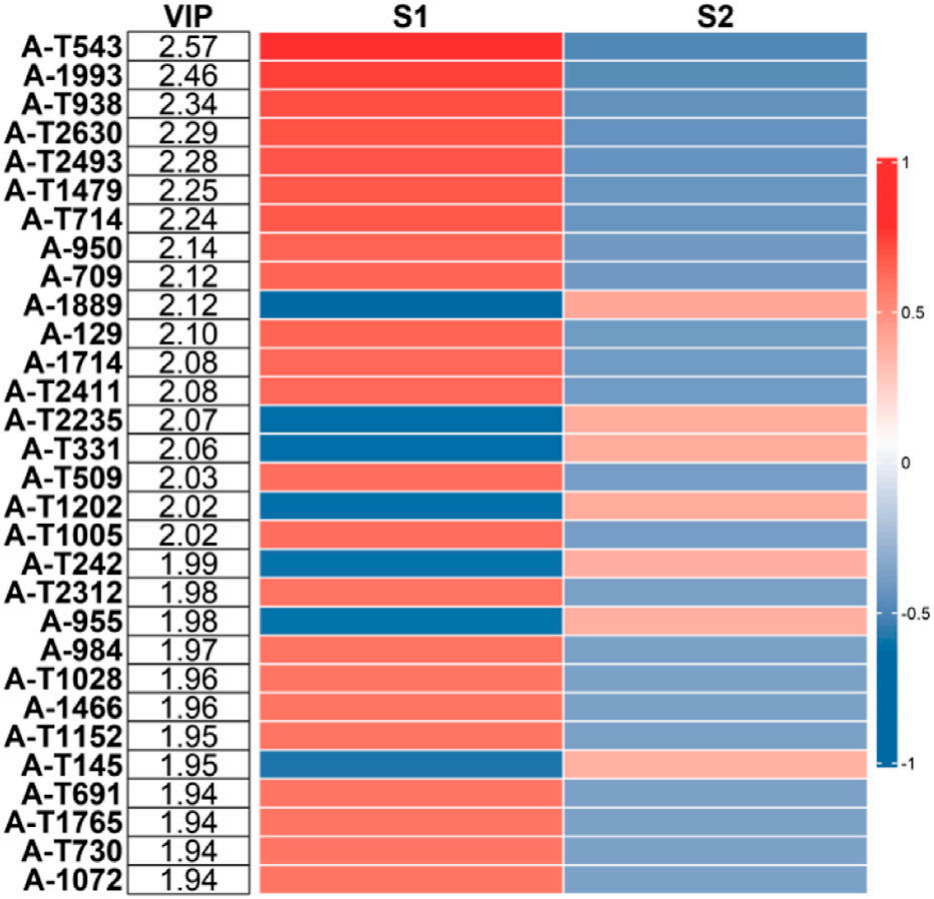
Heatmap of the top 30 metabolites ranked by variable importance for the projection. Note: VIP, variable importance in projection; Change: comparison between the intrinsic and idiosyncratic DILI groups, indicating upregulation or downregulation of metabolites in groups with different pathogenic mechanisms of DILI. A darker color represents a greater magnitude of change.

Based on the aforementioned PLS-DA model, biomarkers with variable importance in projection (VIP) scores greater than 1 were selected. Among the top 30 metabolites ranked by VIP score, the following level 1 and level 2 metabolites:Alanyl-Glycine,N2-Acetyl-L-Cystathionine (level 2a), Isomer 1 of 5-Hydroxyindoleacetic acid (level 2a) and Isomer 1 of 5-Hydroxyindoleacetic acid (level 2a) were identified as exhibiting significant importance in the model. A heatmap depicting these key metabolites according to their VIP scores is provided in Figure 2. The permutation test results (R^2^Y = 0.842, Q^2^ = 0.357) demonstrate that the model exhibits a good fit to the data and possesses a clear and acceptable predictive capability.

#### 2.6.2 Univariate statistical analysis

**FIGURE 3:**
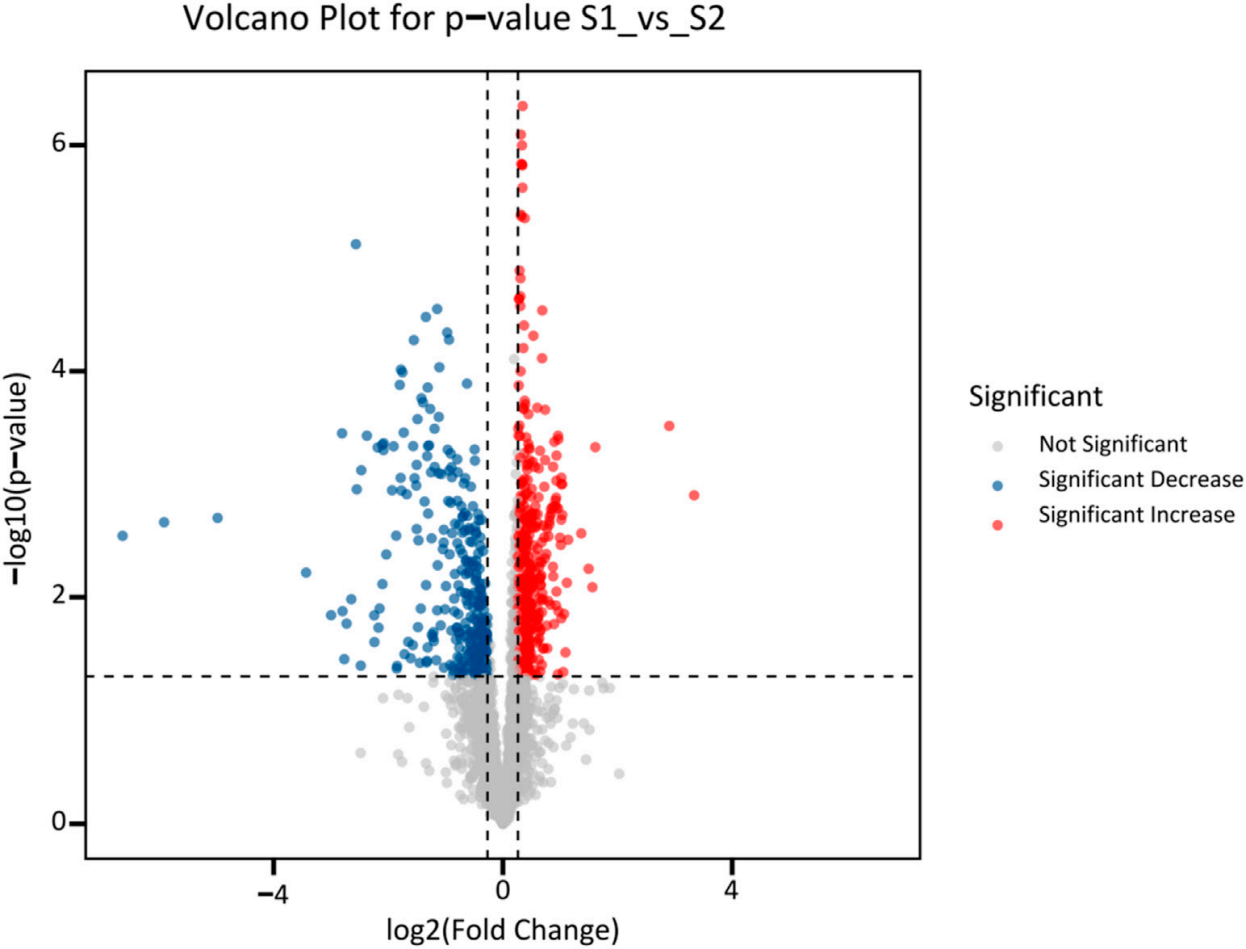
Volcano plot showing significantly differential metabolites in intrinsic versus idiosyncratic DILI.

**FIGURE 4:**
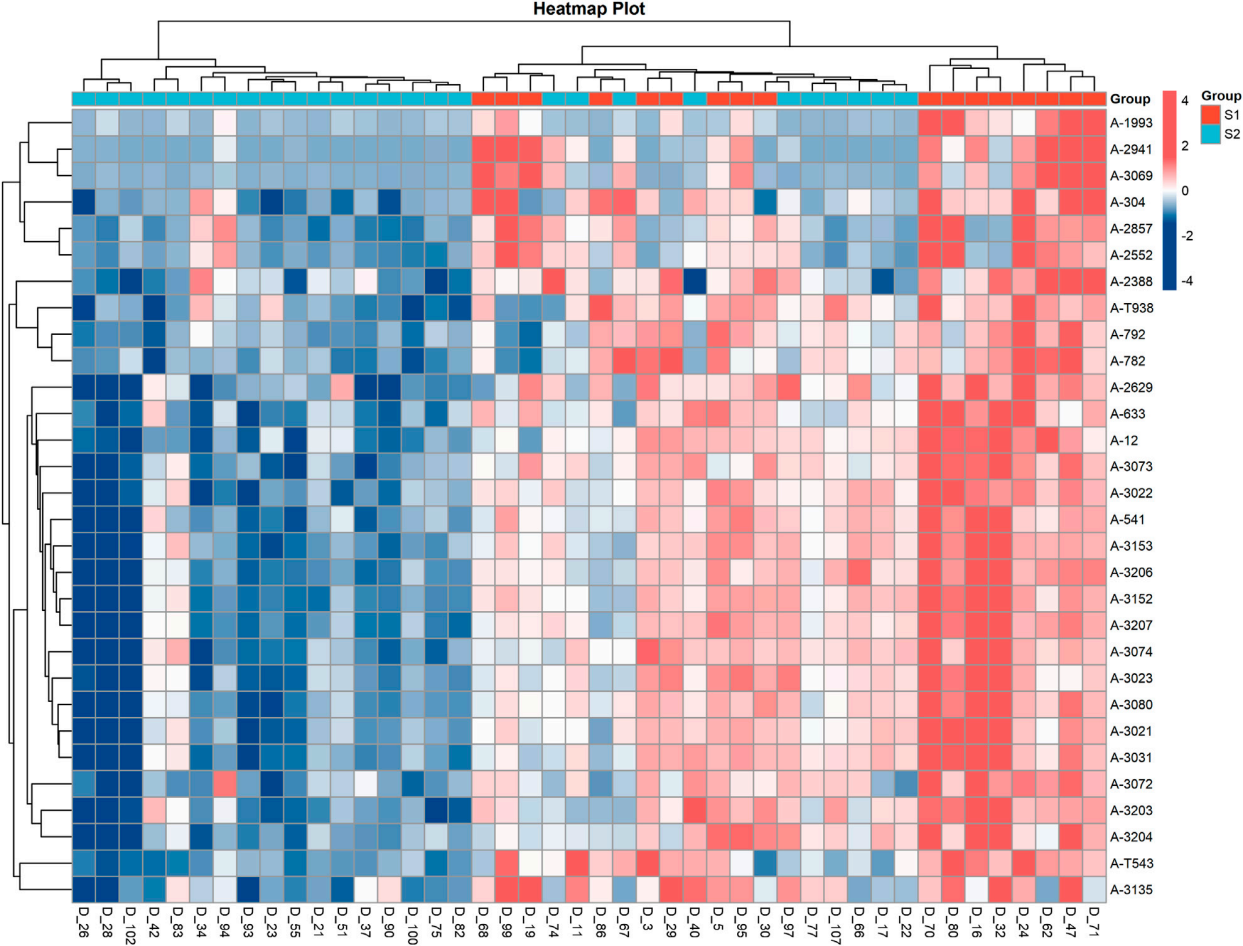
Heat maps of differential metabolites across all samples. Note: VIP (Variable Importance Projection) indicates variable significance. Variation: The comparison between intrinsic and specific groups, where darker colors indicate greater variation.

The volcano plot illustrating differential analysis of all detected metabolites is shown in Figure 3. A total of 723 metabolites were identified as differentially expressed. Compared with the idiosyncratic group, 416 metabolites were significantly upregulated (FC > 1.2, P < 0.05; red dots) and 307 were significantly downregulated (FC < 0.83, P < 0.05; blue dots) in the intrinsic group. Among these, 75 differential metabolites were identified at level 1, and 99 were identified at level 2, based on database matching. The identification results of significantly altered metabolites between the two groups are summarized in Figure 4 and Table 1. A complete list of all 723 statistically significant differential metabolites (416 upregulated and 307 downregulated in intrinsic DILI compared to idiosyncratic DILI) identified by univariate analysis (FC > 1.2 or <0.83, with a raw P-value <0.05) is provided in Supplementary Table S1. To present core results concisely, Table 1 in the main text lists only the top 15 upregulated and downregulated first- and second-level identified metabolites.

#### 2.6.3 Screening of biomarkers for intrinsic and idiosyncratic DILI mechanisms

To identify the most promising potential biomarkers distinguishing intrinsic from idiosyncratic DILI, we compared the results derived from partial least squares-discriminant analysis (PLS-DA) and volcano plot analysis.

The heat map of the screening results from multivariate statistical analysis shows that among the top 20 metabolites with VIP status, the primary and secondary metabolites identified are combined with the screening criteria of” 1.4 univariate statistical analysis “to select the top 15 significant metabolites among the upregulated and downregulated metabolites in the two groups of samples.

From these, the common metabolite significant in both analyses—Alanyl-Glycine—was selected. Receiver operating characteristic (ROC) analysis performed using MetaboAnalyst (http://www.metaboanalyst.ca/) yielded an AUC of 0.874, with a sensitivity of 0.9 and specificity of 0.8.

Based on the multivariate statistical analysis, a heatmap of the top 30 VIP metabolites was generated, from which level 1 and level 2 metabolites were identified. These were combined with the screening criteria of “1.4 univariate statistical analysis” to select the top 15 significant metabolites among the upregulated and downregulated metabolites in the two groups of samples.: Alanyl-Glycine (level 1) (level 1),N2-Acetyl-L-Cystathionine (level 2a), Isomer 1 of 5-Hydroxyindoleacetic acid (level 2a) and Isomer 1 of 5-Hydroxyindoleacetic acid (level 2a). ROC analysis was performed to evaluate the diagnostic potential of these candidate biomarkers.

Researchers performed ROC analysis to evaluate the specific biomarkers identified through the aforementioned statistical approaches. Using MetaboAnalyst, several machine learning methods—including support vector machine (SVM), multiple regression, linear regression, and random forest—were employed to develop diagnostic models for distinguishing intrinsic from idiosyncratic hepatotoxicity.

These models were systematically compared based on key performance metrics, such as sensitivity, specificity, accuracy, precision, F1-score, and AUC-ROC with 95% confidence intervals (Supplementary Table S2). All models exhibited AUC-ROC values exceeding 0.89 and accuracy levels ranging from 0.864 to 0.909, demonstrating that metabolomic feature-based machine learning models offer reliable diagnostic utility for discriminating between the two DILI subtypes. Among them, the SVM model showed the most balanced performance across metrics, whereas the PLS-DA model also achieved notable AUC-ROC results.

**FIGURE 5:**
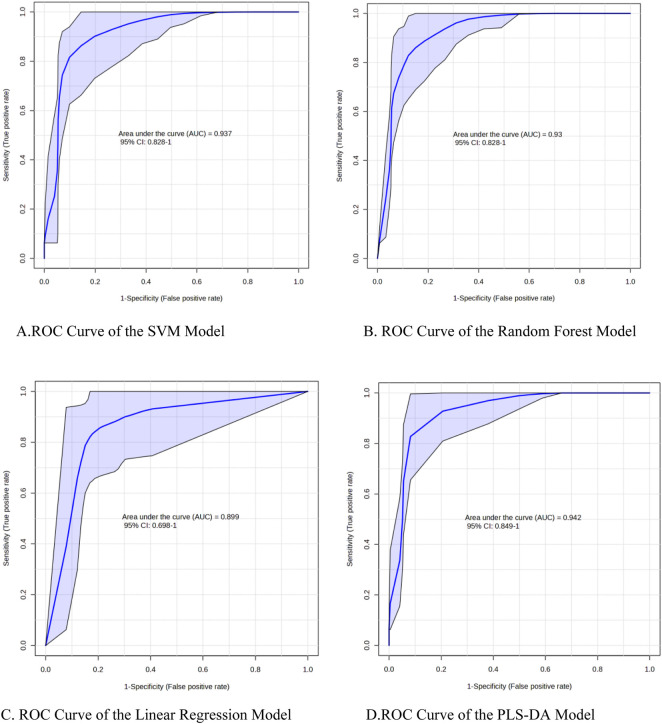
ROC curves of different machine learning models. **(A)** ROC curve of the SVM model **(B)**. ROC curve of the random forest model. **(C)** ROC curve of the linear regression model **(D)**. ROC curve of the PLS-DA model.

Therefore, we propose that the four metabolites identified here—endowed with high diagnostic performance—represent promising candidate biomarkers (see Figure 5). Under the multiple regression framework, internal cross-validation within the training set indicated excellent model tuning and robustness, yielding an AUC of 0.983. Furthermore, holdout validation confirmed the model’s predictive reliability, with an AUC of 0.935.

#### 2.6.4 Metabolic pathway analysis of differential metabolites

To ensure the reliability and interpretability of pathway analysis, only 174 first- and second-level identified differential metabolites obtained from univariate statistical analysis were used for metabolic pathway analysis.

**FIGURE 6:**
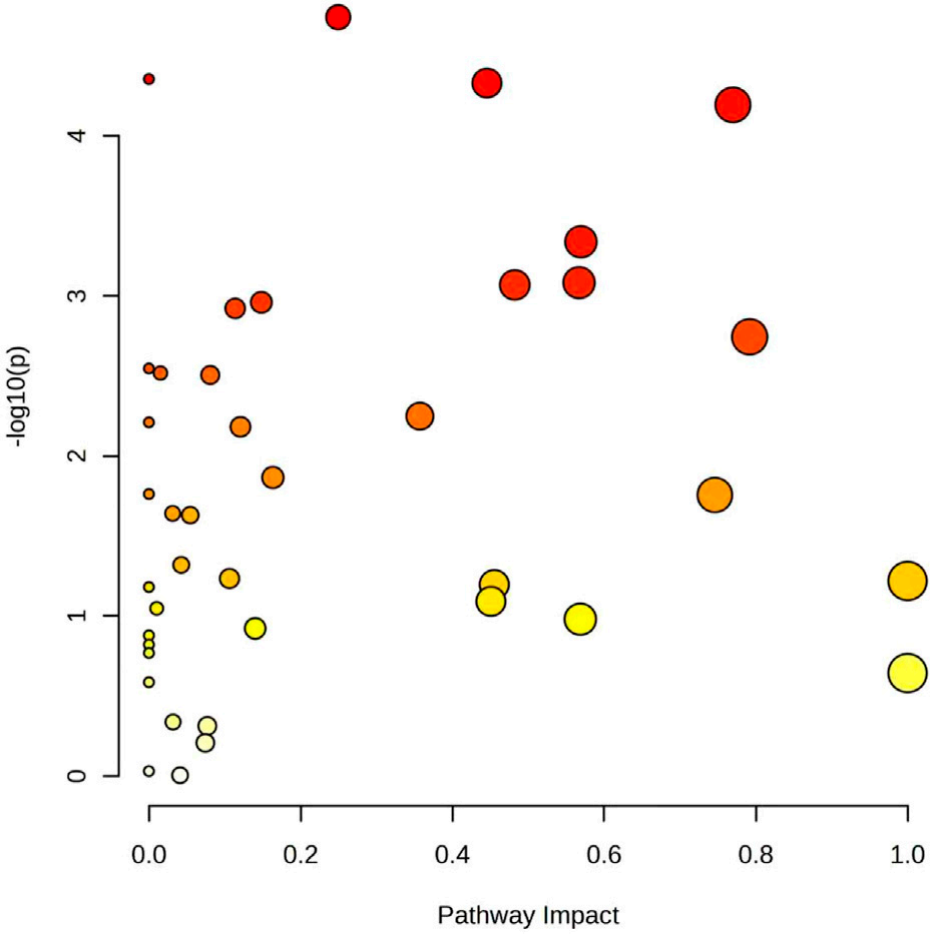
Bubble plot of metabolic pathway analysis of level 1 and level 2 metabolites differing between the intrinsic and idiosyncratic drug-induced liver injury (DILI) groups.

The results revealed that the pathways most considerably affected in distinguishing intrinsic from idiosyncratic DILI mainly involved Tryptophan metabolism, Glycine, serine and threonine metabolism, Cysteine and methionine metabolism, and Arginine and proline metabolism, all of which showed significant metabolic disturbances (Figure 6).

In Figure 6, each bubble represents a specific metabolic pathway. The size of the bubble corresponds to the pathway impact score, while the color intensity indicates the statistical significance of the pathway, with darker colors representing higher significance. The findings suggest that disruptions in amino acid metabolism networks may play a crucial role in differentiating between intrinsic and idiosyncratic DILI.

#### 2.6.5 Sample size sufficiency and statistical power

Given the cohort size, a post-hoc power analysis was conducted to evaluate the statistical robustness of our findings. This analysis was based on the large effect sizes (Cohen’s d) of the key differential metabolites identified in this study: Alanyl-Glycine (level 1) (level 1),N2-Acetyl-L-Cystathionine (level 2a), Isomer 1 of 5-Hydroxyindoleacetic acid (level 2a), and Isomer 1 of 5-Hydroxyindoleacetic acid (level 2a). The Cohen’s d values for these metabolites, calculated using the Psychometrica online tool, were 1.629, 1.32, 1.201, and 1.521, respectively. Using the most conservative value (d = 1.201) as input for G*Power software (version 3.1) with an alpha level of 0.05 and the actual sample sizes (S1 = 17, S2 = 27), the achieved statistical power was 0.966. This result demonstrates that the study had >96% power to detect large effects (d > 0.8), confirming that the sample size was sufficient to identify the robust metabolic differences reported in the following sections.

#### 2.6.6 Incorporating patient sex into the machine learning model

Given the observed significant difference in sex distribution within our study cohort and the potential influence of patient sex on distinguishing between intrinsic and idiosyncratic drug-induced liver injury (DILI), we included “patient sex” as an important binary clinical feature. Sex was encoded as a binary variable, with “0” representing female and “1” representing male. Together with the four previously identified differential metabolites, we incorporated the sex variable into machine learning models. The results showed that the incorporation of sex further enhanced the machine learning model’s performance.

#### 2.6.7 Sensitivity analysis

“An important consideration in our study is the inclusion of eight patients with DILI attributed to anticancer chemotherapeutic agents. Given that cancer itself can profoundly alter the host metabolome, we acknowledge the potential for confounding in our analysis. Our sensitivity analysis, which excluded these patients, demonstrated that the core metabolic signature distinguishing intrinsic from idiosyncratic DILI remained robust:the PLS-DA model still showed clear separation, and the key biomarkers retained high diagnostic power with an AUC of 0.863. This suggests that the metabolic disparities we identified are primarily driven by the mechanism of liver injury rather than the presence of an underlying malignancy. The chart can be found in the Supplementary Material 2.

## 3 Discussion

The pathogenesis of drug-induced liver injury (DILI) is complex and has not been fully elucidated. It is conventionally categorized into intrinsic (dose-dependent and predictable) and idiosyncratic (unpredictable and highly variable among individuals) types (Keshari et al., 2025). In recent years, the concept of “indirect” DILI has been proposed, suggesting that some drugs may induce or exacerbate liver injury through their pharmacological effects rather than through intrinsic toxicity or immunogenicity, thereby altering the physiological state of the body (European association for the study of the liver, 2019). However, this proposed mechanism currently lacks sufficient clinical and experimental support, and its distinction from idiosyncratic DILI remains ambiguous, making clear differentiation challenging (Li et al., 2024; Fontana et al., 2023). Therefore, this study focuses specifically on comparing the mechanisms underlying intrinsic and idiosyncratic DILI.

The core challenges in this field are primarily reflected in the following aspects: First, there is a lack of objective diagnostic tools, as current clinical biochemical indicators and pathological features are insufficient to reliably differentiate between DILI caused by distinct mechanisms (intrinsic, idiosyncratic). Second, the classification of DILI mechanisms for specific drugs largely relies on empirical consensus among experts rather than well-validated objective biomarkers. Finally, it remains unclear whether fundamental differences in pathophysiological mechanisms exist between intrinsic and idiosyncratic DILI, owing to the absence of in-depth and systematic experimental data. Therefore, elucidating the various mechanisms underlying drug-induced liver injury and identifying corresponding biomarkers are of crucial importance for the scientific prevention, diagnosis, and treatment of DILI.

This study represents the first application of HP-CIL LC-MS-based metabolomics to investigate alterations in endogenous small molecule metabolites in the serum of patients with intrinsic versus idiosyncratic drug-induced liver injury (DILI). The PLS-DA model demonstrated clear separation between the two groups. We subsequently integrated the results from both PLS-DA and volcano plot analyses to identify metabolites that showed consistent significance across both methods. These selected common metabolites were then used to construct diagnostic models for discriminating between intrinsic and idiosyncratic DILI using multiple machine learning approaches implemented in MetaboAnalyst. Ultimately, four differential metabolites:Alanyl-Glycine (level1),N2-Acetyl-L-Cystathionine (level 2a), an Isomer of 5-Hydroxyindoleacetic Acid, and Isomer 1 of 5-Hydroxyindoleacetic acid (level 2a)—were identified as potential biomarkers for distinguishing the two types of DILI. The initial machine learning model, which did not include sex as a feature, demonstrated that the diagnostic model constructed from the combination of four metabolites already exhibited strong discriminatory performance. This study further highlights the important influence of patient sex on model performance. Analysis of the clinical cohort revealed a significant difference in sex distribution between the two groups—the intrinsic DILI group was 76.5% female, whereas the idiosyncratic DILI group was 77.8% male (χ^2^ = 12.51, P < 0.001). After incorporating patient sex as a binary feature into the original model, the results showed a consistent further improvement in the machine learning model’s performance, underscoring the value of sex as an informative variable in subtype discrimination.

Metabolic pathway analysis of the differentially expressed metabolites revealed that the most impacted pathways included Tryptophan metabolism, Glycine, serine and threonine metabolism, Cysteine and methionine metabolism, and Arginine and proline metabolism, suggesting that disruptions in amino acid metabolism may play a critical role in differentiating between intrinsic and idiosyncratic DILI (Xu et al., 2012; Xu et al., 2018).

The Alanyl-Glycine (level 1) screened in this study is a dipeptide formed by the linkage of alanine and glycine via a peptide bond, which has been identified in human urine. It is a degradation product of both endogenous and exogenous proteins (Wishart et al., 2022). Dipeptides play essential roles in nutritional and biological functions, and recent studies have reported their potential as biomarkers for the diagnosis and classification of various diseases (Li et al., 2019; Wu et al., 2013; Ozawa et al., 2020). Many researchers have identified differential dipeptide metabolites in different disease models using untargeted metabolomic approaches (Hou et al., 2021).

After cellular uptake, a portion of dipeptides is hydrolyzed by cytoplasmic peptidases into free amino acids, which are then utilized for cellular metabolic synthesis or transported into systemic circulation via basolateral amino acid transport systems. Another fraction of dipeptides, resistant to hydrolytic enzymes, can be transported intact into the circulation via basolateral peptide transporters, after which they are degraded into amino acids by soluble proteases in the plasma (Xu et al., 2019; Heidenreich et al., 2021; Rossignol et al., 2021).

In intrinsic drug-induced liver injury (DILI), the elevation of Alanyl-Glycine (level 1) may be closely associated with its hydrolysis into individual amino acids—alanine and glycine—which subsequently enter their respective metabolic pathways.

Glycine, a key component of glutathione (GSH), contributes to the detoxification process. In acetaminophen-induced intrinsic hepatotoxicity, CYP-mediated metabolism generates reactive metabolites accompanied by depletion of GSH, accelerating the formation of reactive oxygen and nitrogen species in necrotic hepatocytes. Impaired liver function or necrosis reduces hepatic amino acid metabolic capacity, leading to the release of intracellular amino acids into the bloodstream, thereby increasing plasma glycine levels (Yu et al., 2017; Wang et al., 2017). On the other hand, alanine serves as an important gluconeogenic amino acid. Within the glucose-alanine cycle, it can be converted into glucose via gluconeogenesis. DILI impairs gluconeogenesis, disrupting glucose synthesis and ultimately resulting in significantly elevated alanine levels (Wang et al., 2017). Additionally, DILI enhances glycolytic metabolism. As reported by Huo et al., sodium valproate—an agent known for intrinsic hepatotoxicity—increased pyruvate and lactate levels in DILI patients, indicating enhanced anaerobic metabolism, which is often accompanied by a rise in alanine (Huo et al., 2014). Therefore, the increase in Alanyl-Glycine (level 1) may reflect concurrent metabolic disturbances of both glycine and alanine in the context of intrinsic DILI. This process not only illustrates the metabolic relationship between the dipeptide and its constituent amino acids, but also suggests that Alanyl-Glycine (level 1) (level 1) could serve as a meaningful biomarker indicating overall dysregulation of amino acid metabolism in liver injury.

N2-Acetyl-L-cystathionine is a sulfur-containing amino acid derivative associated with human inborn errors of metabolism such as cystathioninuria (Wishart et al., 2022). It is likely an acetylated product of cystathionine, which serves as a key intermediate in the methionine metabolic pathway and plays a central role in the transsulfuration pathway for cysteine synthesis (Zheng and Conrad, 2025). In the context of intrinsic hepatotoxicity induced by agents such as acetaminophen (APAP), liver injury is initiated through rapid depletion of glutathione (GSH). Metabolomic studies have shown that GSH depletion leads to the exhaustion of precursor metabolites—including cysteine and methionine—as part of the cellular compensatory response to APAP-induced oxidative stress (Kralj et al., 2021). In an attempt to restore GSH levels, the transsulfuration pathway may be upregulated, resulting in the accumulation of cystathionine. This, in turn, drives the elevation of its acetylated derivative,N2-Acetyl-L-Cystathionine (level 2a). Therefore, the elevation ofN2-Acetyl-L-Cystathionine (level 2a) may serve as a downstream metabolic marker indicative of enhanced transsulfuration activity in hepatocytes responding to glutathione depletion during intrinsic drug-induced liver injury.

Amino acid metabolism, particularly involving tryptophan, has been documented in previous studies as a common biomarker associated with hepatotoxicity (Zhao et al., 2019). As an essential amino acid, tryptophan participates in various biosynthetic pathways, and its metabolic disruption is implicated in the pathogenesis of liver dysfunction.

No studies have been reported to date regarding Isomer 1 of 5-Hydroxyindoleacetic acid (level 2a) or Isomer 1 of 5-Hydroxyindoleacetic acid (level 2a) in the context of DILI research.

Several metabolomic studies related to drug-induced liver injury (DILI) have been published. Quintás et al. (2021) conducted a serum metabolomic analysis of patients with three DILI phenotypes (hepatocellular, cholestatic, and mixed) and revealed that free and conjugated bile acids, as well as glycerophospholipids, were the most relevant metabolic features associated with DILI phenotypes. Xie et al. (2021) applied ultra-high-performance liquid chromatography coupled with triple quadrupole mass spectrometry to profile serum metabolites from DILI patients with varying severity levels. They identified deoxycholic acid, taurodeoxycholate, and glycodeoxycholate as biomarkers distinguishing severe from non-severe DILI. García-Cañaveras et al. (2016) employed an untargeted metabolomics approach based on liquid chromatography–mass spectrometry to analyze the metabolic profiles of HepG2 cells treated with drugs known to cause liver injury through different mechanisms. Their findings indicated that glutathione-related metabolites, the lysophospholipid/phospholipid ratio, and intermediates of fatty acid β-oxidation served as key metabolic markers differentiating mechanisms such as oxidative stress, phospholipidosis, and steatosis.

This study is the first to employ HP-CIL LC–MS based metabolomics to investigate metabolic differences between intrinsic and idiosyncratic DILI, with the aim of identifying biomarkers capable of predicting these two DILI subtypes. By integrating metabolomic data with clinical variables and applying machine learning strategies, we preliminarily identified potential biomarkers associated with the mechanisms of intrinsic and idiosyncratic DILI, and further developed a predictive model for distinguishing the two subtypes. Considering the limited sample size, 60% of the data from both the intrinsic and idiosyncratic DILI groups were used as the training set during the biomarker screening phase, while the remaining data served as the test set. Under various machine learning strategies, the selected specific biomarkers were evaluated, and the model demonstrated reliable predictive performance based on the test set results.

Following the cluster analysis, we retrospectively reviewed the clinical characteristics and medication histories of the patients within the third cluster. We found that this cluster likely consists of patients who developed DILI following the use of anticancer chemotherapeutic agents. This discovery provided a crucial explanatory clue.

In recent years, the classification of DILI pathogenesis has been evolving. The traditional intrinsic-idiosyncratic dichotomy is being challenged. As the reviewer speculated, our third cluster might correspond to a newly proposed mechanism—indirect DILI. An authoritative review published in the New England Journal of Medicine in 2019 first systematically introduced this concept, specifying liver injury triggered by the drug’s pharmacological effects themselves, rather than its direct hepatotoxicity or immunogenicity, often manifesting in individuals with underlying liver disease or specific susceptibilities (Hoofnagle and Björnsson, 2019).

Anticancer drugs have traditionally been classified as intrinsic DILI, but recent guidelines and perspectives tend to reclassify many of their cases as indirect (Fontana et al., 2023). This is because their liver injury is often associated with indirect effects caused by cytotoxicity (e.g., immunosuppression, mitochondrial stress) rather than direct membrane dissolution. Regarding the challenge of distinguishing it from Idiosyncratic DILI: As noted in the literature (Fontana et al., 2023), indirect DILI shares similarities in clinical presentation (e.g., dose-independent nature) with idiosyncratic DILI, making practical differentiation difficult. This explains why in our unsupervised cluster analysis, the third cluster, potentially representing indirect DILI, was identified as a distinct metabolic entity rather than being merged into the cluster of idiosyncratic DILI. This suggests that, at the metabolomic level, it may possess a unique background metabolic network perturbation.

This study has several limitations. First, the sample size is relatively small, and future investigations should incorporate larger cohorts to enhance the robustness and generalizability of the findings. Second, the metabolomic analysis focused solely on intrinsic and idiosyncratic DILI without including the proposed “indirect” DILI subtype, primarily due to the current lack of sufficient clinical and experimental evidence supporting its distinct pathological basis and diagnostic criteria, as well as its potential overlap with idiosyncratic DILI in clinical and mechanistic features. In contrast, the intrinsic-idiosyncratic classification has been well established for over 30 years and is widely endorsed in international guidelines, offering a more solid and consensus-driven framework. Finally, the functional roles and dynamic changes of the identified biomarkers during the progression of DILI require further validation through additional mechanistic and longitudinal studies.

In summary, we applied a metabolomics approach to characterize both drug subtypes and cases of DILI with well-established mechanisms, encompassing intrinsic and idiosyncratic types. This study revealed distinct metabolic profiles between intrinsic and idiosyncratic DILI and, through integration with machine learning strategies, identified potential biomarkers associated with these two mechanistic subtypes. The PLS-DA model based on serum metabolomics demonstrated promising clinical predictive value for ATB-DILI, though further validation in larger and more targeted studies is warranted. These findings may provide valuable insights for the diagnosis and treatment of DILI, as well as new perspectives on its pathogenic mechanisms from a metabolomic standpoint.

## Data Availability

The metabolomics dataset has been deposited in the MetaboLights repository under accession number MTBLS13327.
