# Supplementary material for: Machine learning-assisted analysis of serum metabolomics for identifying biomarkers in intrinsic and idiosyncratic drug-induced liver injury
Source: Front Pharmacol. 2026 Feb 27;16:1727462. doi: 10.3389/fphar.2025.1727462 (PMC12984056; doi:10.3389/fphar.2025.1727462)
Supplement: Supplementary file 3 [file Supplementaryfile3.docx]

**Supplementary Material 3**.

| Machine Learning Model | Support Vector Machine | Random Forest | Logistic Regression | PLS-DA |
| --- | --- | --- | --- | --- |
| Metabolites Only（AUC-ROC） | 0.937 | 0.93 | 0.899 | 0.942 |
| Metabolites + Sex（AUC-ROC） | 0.958 | 0.959 | 0.902 | 0.951 |

Table 2: Comparison of Model Performance (Metabolites Only vs. Metabolites + Sex)


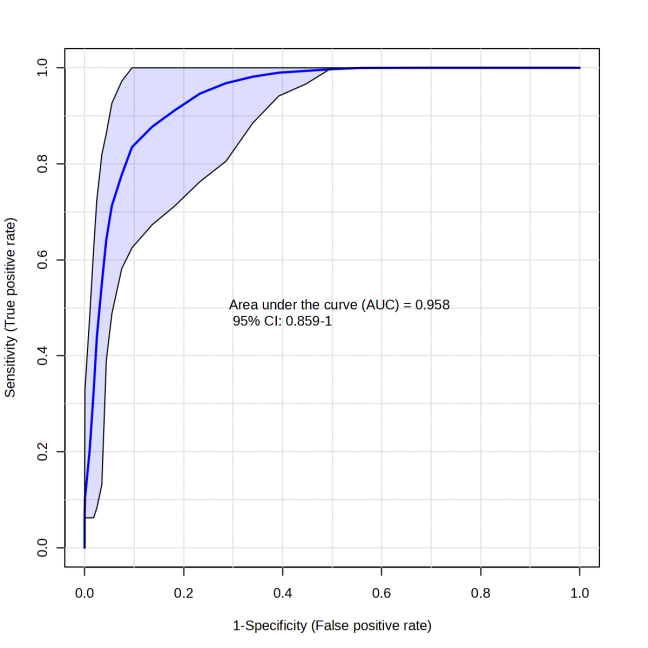

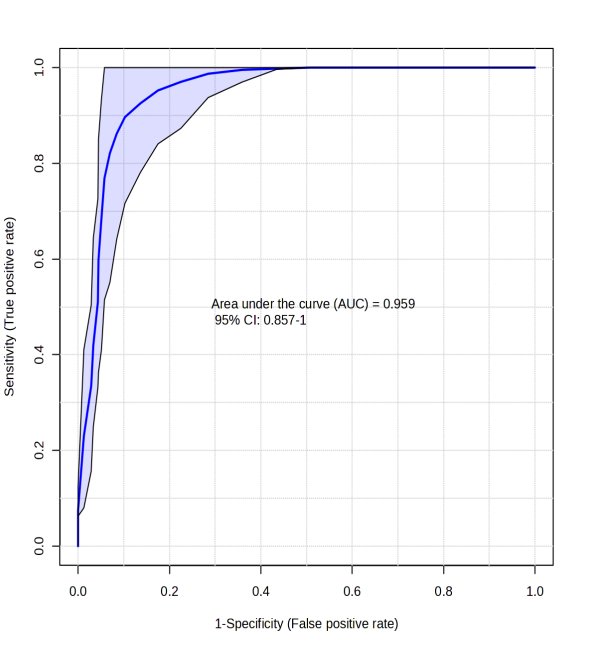


A.ROC Curve of the SVM Model B. ROC Curve of the Random Forest Model


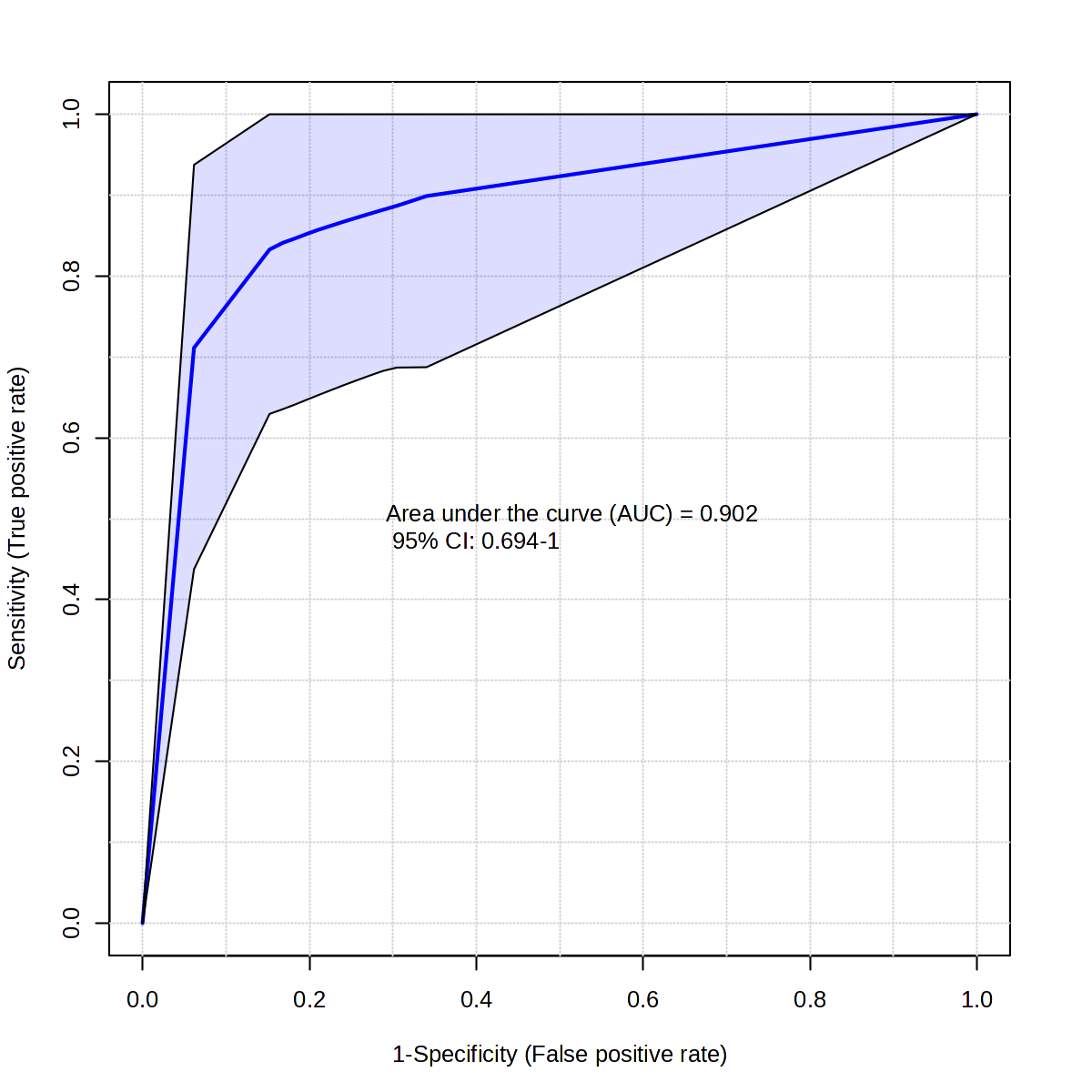

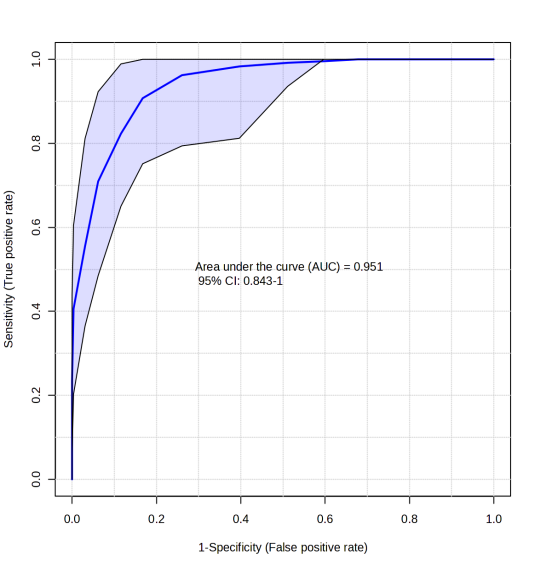


C. ROC Curve of the Linear Regression Model D.ROC Curve of the PLS-DA
