# Supplementary material for: Machine learning-assisted analysis of serum metabolomics for identifying biomarkers in intrinsic and idiosyncratic drug-induced liver injury
Source: Front Pharmacol. 2026 Feb 27;16:1727462. doi: 10.3389/fphar.2025.1727462 (PMC12984056; doi:10.3389/fphar.2025.1727462)
Supplement: Supplementary file 4 [file Supplementaryfile2.docx]

Supplementary Material 2：Sensitivity Analysis of Intrinsic versus Idiosyncratic DILI after Excluding Patients with Anticancer Chemotherapy-Induced Liver Injury (n=36)


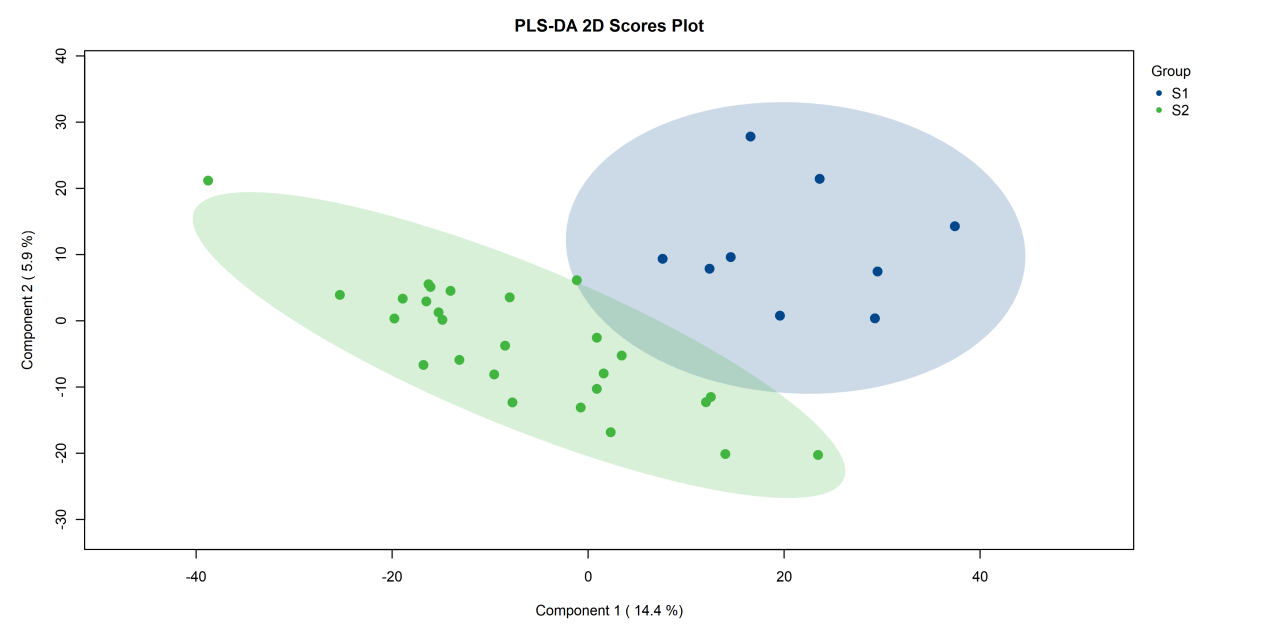


**Fig S1** PLS-DA Analysis of the Intrinsic DILI Group versus the Idiosyncratic DILI Group


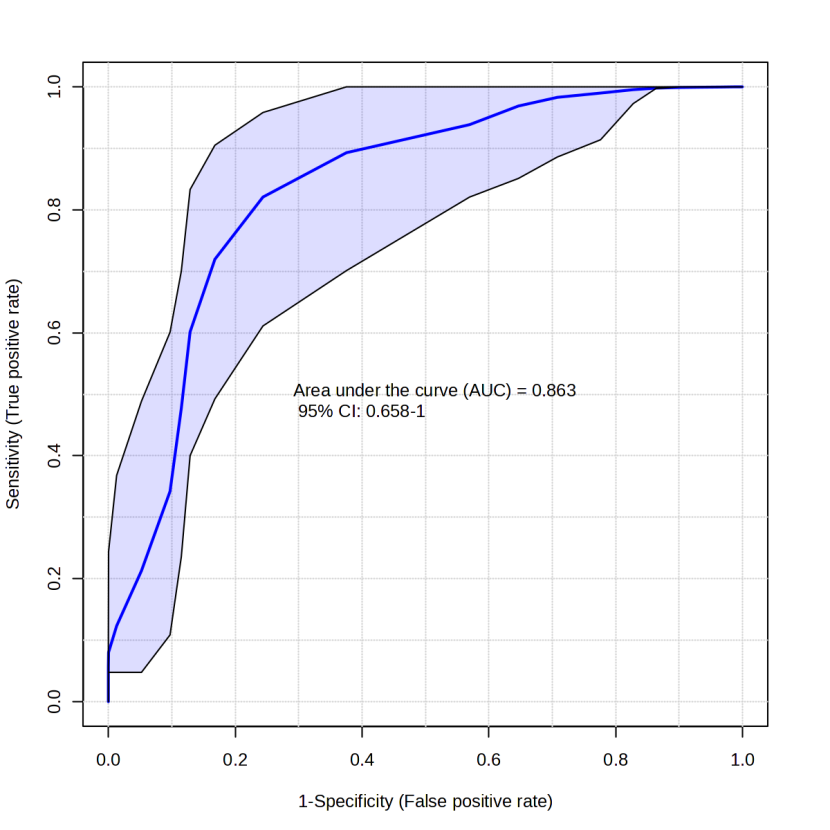


**Fig S2** ROC Curves of the PLS-DA Model
