# Supplementary material for: Machine learning-assisted analysis of serum metabolomics for identifying biomarkers in intrinsic and idiosyncratic drug-induced liver injury
Source: Front Pharmacol. 2026 Feb 27;16:1727462. doi: 10.3389/fphar.2025.1727462 (PMC12984056; doi:10.3389/fphar.2025.1727462)
Supplement: Supplementary file 5 [file Supplementaryfile4.docx]

**Supplementary Material 4**

| **Model** | **Sensitivity**  **(Recall)** | **Specificity** | **Accuracy** | **Precision** | **F1-Score** | **AUC-ROC**  **（95%CI）** |
| --- | --- | --- | --- | --- | --- | --- |
| Support Vector Machine | 0.928 | 0.882 | **0.909** | **0.926** | **0.926** | 0.937（0.828-1.000） |
| PLS-DA | 0.893 | 0.875 | 0.886 | 0.926 | 0.909 | 0.942 (0.849 - 1.000) |
| Logistic Regression | 0.920 | 0.789 | 0.864 | 0.852 | 0.885 | 0.899(0.698 - 1.000) |
| Random Forest | **0.893** | **0.875** | **0.886** | **0.926** | **0.909** | **0.930 (0.828-1.000)** |

Supplementary Table S2. Performance Metrics of Machine Learning Models for Discriminating Intrinsic vs. Idiosyncratic DILI Subtypes
